# True equilibrium measurement of transcription factor-DNA binding affinities using automated polarization microscopy

**DOI:** 10.1038/s41467-018-03977-4

**Published:** 2018-04-23

**Authors:** Christophe Jung, Peter Bandilla, Marc von Reutern, Max Schnepf, Susanne Rieder, Ulrich Unnerstall, Ulrike Gaul

**Affiliations:** Gene Center and Department of Biochemistry, Center for Integrated Protein Science Munich (CIPSM), Ludwig-Maximilians-Universität München, Feodor-Lynen-Strasse 25, 81377 München, Germany

## Abstract

The complex patterns of gene expression in metazoans are controlled by selective binding of transcription factors (TFs) to regulatory DNA. To improve the quantitative understanding of this process, we have developed a novel method that uses fluorescence anisotropy measurements in a controlled delivery system to determine TF-DNA binding energies in solution with high sensitivity and throughput. Owing to its large dynamic range, the method, named high performance fluorescence anisotropy (HiP-FA), allows for reliable quantification of both weak and strong binding; binding specificities are calculated on the basis of equilibrium constant measurements for mutational DNA variants. We determine the binding preference landscapes for 26 TFs and measure high absolute affinities, but mostly lower binding specificities than reported by other methods. The revised binding preferences give rise to improved predictions of in vivo TF occupancy and enhancer expression. Our approach provides a powerful new tool for the systems-biological analysis of gene regulation.

## Abstract (web summary)

Methods to measure selective transcription factor-DNA binding often lack sensitivity and are not performed in solution. Here the authors develop a method to perform fluorescence anisotropy measurements of transcription factor-DNA binding energies with high sensitivity and throughput.

## Introduction

The key event in the spatio-temporal control of gene expression is the binding of transcription factors (TFs) to regulatory DNA. While ChIP-Seq and related methods have proven useful in mapping TF binding to DNA genome-wide in vivo, they have significant limitations, among them low spatial and quantitative resolution, and the likelihood of capturing substantial unspecific or non-functional binding. Thus, measuring TF-DNA interactions accurately in a controlled in vitro environment remains a highly useful, complementary approach. The TF binding preferences derived from such techniques, in the form of position-weight matrices (PWMs), can subsequently be used to predict binding sites in the genome, and in this fashion have proven to be vital tools in reconstructing and modeling gene regulatory networks^1,2^. In eukaryotes, the binding of TFs to DNA and the resulting occupancy landscape is thought to be well described by equilibrium thermodynamics, and the ideal method to quantify TF-DNA interactions will therefore approximate equilibrium conditions as closely as possible. This means that the measurement should take place in solution, with no immobilization of interaction partners or other mechanical or chemical interferences that might affect the properties of the binding reaction^3^. The assay should be sensitive enough to accurately capture both very high-affinity binding and comparatively weak binding, i.e., washing and other filtering steps should be avoided. Finally, the assay should be scalable such that the measurements can be performed efficiently for large numbers of TFs and DNA sequences.

None of the currently available methods fulfill all these criteria (for overview see Supplementary Table 1). The most widely used high-throughput methods measure binding in non-equilibrium: protein binding microarrays (PBM)^4^ have increased their throughput, but suffer from low sensitivity because of stringent wash requirements, causing loss of weak binders. High-throughput systematic evolution of ligands by exponential enrichment (HT-SELEX)^5,6^ allows probing a large sequence space, and SELEX-Seq^7^ can even be used to determine their relative affinities. However, both techniques require a resin- or filter-based selection step (including washing) that introduces bias. Finally, bacterial one-hybrid (B1H)^8^ has allowed the characterization of the binding specificities of hundreds of transcription factors, but is based on a strict bacterial survival selection. All these techniques thus allow the testing of a large sequence space, but include stringent washing or selection steps that exclude all but the very strongest binders. Therefore, while they have proven very useful and accurate in establishing consensus binding motifs de novo, they typically arrive at overly specific motifs. In practice, these highly specific binding motifs are often blurred by the addition of “pseudo-counts”, thus artificially introducing weaker binding, to permit modeling of experimental data or predicting expression patterns. In addition, these methods critically rely on computational algorithms to identify motifs and model binding specificities from the sequences of the binders. Different approaches to the problem have been developed, and the appearance and information content of a binding motif strongly depends on this analysis step^9,10^.

In other experimental binding assays, such as surface plasmon resonance (SPR)^11^, mechanically induced trapping of molecular interactions (MITOMI)^12^, high-throughput sequencing-fluorescent ligand interaction profiling (HiTS-FLIP)^13^, and selective microfluidics-based ligand enrichment followed by sequencing (SMiLE-Seq)^14^, the binding events take place on a thin surface, rather than in solution. While occasionally described as operating in equilibrium^12^, all such surface-bound assays have significant limitations^15,16^: since on a surface the number of molecules participating in the binding event is much smaller, the assays tend to be less sensitive when measuring very low dissociation constants (*K*_D_s), where protein concentrations are necessarily low. Since the fluorescent background is higher on a surface than in solution, adding noise, this in practice limits surface methods to measure *K*_D_s in the nM range^12,13^ (Supplementary Fig. 3c); very high-affinity binding in the pM range, as is commonly found in TF-DNA interactions, is thus not accurately captured. Moreover, binding to thin surfaces can lead to steric hindrance, unspecific adsorption, and reduced molecular activity, making it difficult to accurately quantify weak binding. Washing steps similarly curtail the capture of weaker binding, and the immobilization of reaction partners on a surface can alter the interaction properties. Finally, since these methods use direct and not competitive titration, the active TF concentration cannot be determined. Yet other assays, such as DNase footprinting^17^, electrophoretic mobility shift assay (EMSA)^18^, and micro-scale thermophoresis (MST)^19^ do work in equilibrium and solution, but are rather low in throughput.

To overcome the limitations of all these approaches, we have devised a method that measures TF-DNA binding affinities in equilibrium and in solution at relatively large scale. The method is based on fluorescence anisotropy (FA), which is widely used for determining the binding affinities between proteins and their ligands, and has the advantage of measuring the interaction strength optically and without interference with the binding event. We have developed this method into high performance FA (HiP-FA) such that it reliably covers a much wider dynamic range of absolute binding affinities (10 pM < *K*_D_ < 10 μM) with binding partners of equal molecular weight, and scaled the assay such that a sufficiently large sequence space can be sampled with modest effort. We validate HiP-FA experimentally using EMSA and MST, and computationally by modeling ChIP-Seq data^20^ and enhancer expression patterns.

## Results

### The HiP-FA assay

**Fig. 1:**
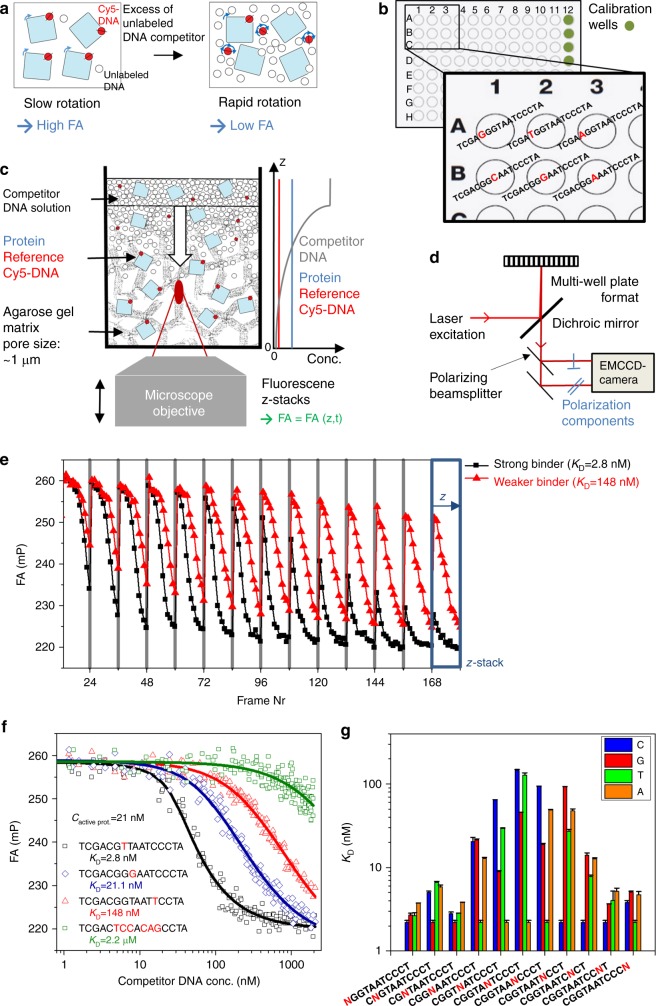
HIP-FA assay and Bcd DNA-binding affinities. **a** Schematic depiction of fluorescence anisotropy (FA) assay. **b** Typical layout of a 96-well plate. Competitor DNA oligomers with different sequences are added on top of the titration wells. The concentration *c*(*z*,*t*) of the competitor DNA is determined by using calibration wells (green) that contain the intercalating dye, Nile Blue (NB), with competitor DNA added on top. **c** Design of gel delivery system for titrating competitor DNA in single wells. Protein and labeled reference DNA are embedded in porous agarose gel. The concentration profiles of TF, dye-reference DNA, and competitor DNA are depicted on the right. FA(*z*,*t*) is measured iteratively in each well, while the competitor DNA is diffusing from the top into the gel matrix. **d** Simplified HIP-FA microscopy setup. **e** FA(*z*,*t*) time trajectories for two titration wells measuring BCD binding to strong (in black) and moderate (in red) DNA competitor. The *z*-stacks are acquired sequentially from the bottom (low DNA concentrations = high FA) to the top (high DNA concentrations = low FA) of the gel. In total, 20 measurement cycles are performed in 7.5 min time intervals. The titration of the protein by the competitor DNA can be monitored by the decrease of FA within a *z*-stack (high to low FA) over time. At *t* = 0, the FA is high, since all labeled reference DNA is bound to the protein; at the end of the measurements, the FA is low, since the labeled reference DNA has been replaced by the unlabeled competitor DNA throughout the gel. The FA(*z*,*t*) decreases faster over time for the strong binder. **f** Representative FA titration measurements for Bcd and fitted curves for strong (black), moderate (blue), weak (red), and very weak (green) binding (mutated bases relative to consensus in red). **g** *K*_D_ values for all 33 possible single-point mutations of the Bcd consensus sequence CGG**TAATCC**CT; the nucleotides marked in bold represent the core binding site. The mutated positions are marked in the sequence (N), columns show *K*_D_s for sequences containing nucleotides C, G, T, and A at that position, as indicated. Error bars represent standard deviation of two replicates

High performance fluorescence anisotropy (HiP-FA) is based on the established fluorescence anisotropy (FA) approach^21^. FA provides a measure of the rotational speed of a fluorescently labeled species, in our case a DNA oligomer. Binding with TF increases the molecular weight and thereby decreases the rotational speed of the labeled DNA oligomer, resulting in increased FA (Fig. 1a). We implemented two crucial improvements over a standard FA setup. First, we carry out a competition experiment using a controlled delivery system within a single well (Fig. 1b, c). TF and fluorescently labeled reference DNA are embedded in a porous matrix and an unlabeled competitor DNA is loaded on top. The competitor DNA forms a spatio-temporal gradient, leading to a dynamically changing FA(*z*,*t*) signal. Second, we use a customized epifluorescence microscope setup, allowing for scoring FA values along the *z*-axis and providing greatly increased sensitivity. Thus, up to 300 data points of a titration curve can be measured in each well of a multi-well plate and, by curve-fitting, both the active protein concentration and the absolute *K*_D_ can be extracted (Fig. 1e, f). The improved sensitivity makes it possible to measure binding between molecules of similar molecular weight, in our case TF protein and its DNA binding sequence.

Our setup consists of a conventional automated wide-field microscope that is modified to accommodate polarized laser light excitation and detection of the two polarization components of the emitted fluorescence using a high numerical aperture (NA) objective and an ultra-sensitive EM-CCD camera (Fig. 1d and Supplementary Fig. 1). These modifications are readily implemented and moderate in cost.

**Fig. 2:**
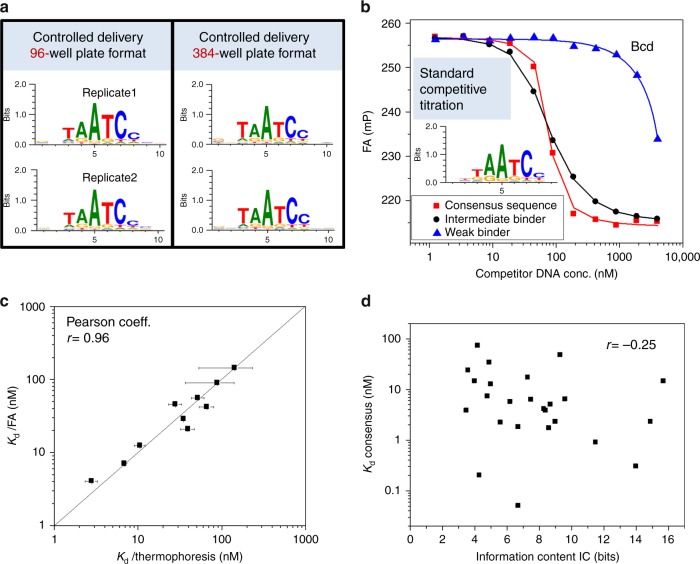
Validation and reproducibility of the HiP-FA method and correlation between the dissociation constants and the information content. **a** Bcd binding motifs obtained from measurements in 96- (left) and 384- (right) well plates using automation. The mean CVs for the *K*_D_s were 5% and 16% for the 96- and 384-well plate formats, respectively. **b** Conventional competitive titration with HIP-FA of three competitor DNAs binding to Bcd with different affinity. The inset shows the Bcd binding motif obtained by conventional competitive titration (average of two replicates). **c** Scatter plot of *K*_D_s obtained by HIP-FA vs. MST, each measured in a competitive assay for BCD-DBD binding to 10 different competitor sequences. Error bars represent standard deviations of two replicate measurements. **d** Scatter plot of consensus sequence *K*_D_ vs. information content (IC) of PWMs for the 26 TFs tested

In the competitive binding assay, TF and dye-labeled reference DNA (Cy5 or BODIPY630) are mixed at fixed concentrations and embedded together in a porous agarose gel. The TF concentration is in molar excess over dye-reference DNA to ensure that all DNA oligomers are bound to the protein. Unlabeled competitor DNA is then added on top of the agarose and, by diffusion, establishes a concentration gradient *c*(*z*,*t*) within the gel, whose shape changes over time and with the position of the focal plane *z* (Fig. 1c and Supplementary Fig. 2). The agarose matrix constitutes a non-interacting aqueous environment and prevents convection, essential for the reproducibility of DNA diffusion between wells. As the competitor DNA diffuses through the matrix, it competes with the dye-reference DNA for binding to the TF, resulting in a dynamically changing FA signal of the dye-reference DNA, FA(*z*,*t*) (Fig. 1e). This allows us to measure, over time, a continuous titration series for a given competitor DNA oligomer within a single well, comprising about 300 individual data points. The *K*_D_ of the dye-reference DNA is explicitly determined by titrating with the same but unlabeled DNA sequence. To determine the competitor concentration *c*(*z*,*t*), which is needed to calculate the TF-DNA dissociation constant, we use separate calibration wells, typically five per plate, where DNA intercalating dye Nile Blue (NB) is incorporated into the gel matrix and *c*(*z*,*t*) is determined by measuring the dynamically changing FA signal of NB, FA_NB_(*z*,*t*) (Supplementary Fig. 2). Each well is usually measured 25 times at 12 different *z*-positions. Single-well measurement of an entire titration curve and competitive binding greatly reduces the amount of required protein (4 or 0.25 pmol of protein per titration curve in 96- or 384-well plate format, Figs. 1b and 2a) and increases fidelity and throughput. The assay can be performed manually, with a coefficient of variation (CV) of <20%, or fully automated using a robotic system, with improved reproducibility (CV < 15%) (Supplementary Data 1). With the controlled delivery system, *K*_D_s can be reliably measured down to 0.5 nM. For extremely high affinities (*K*_D_ < 0.5 nM), we use conventional competitive titration (Fig. 2b and Methods), due to the limitations in accurately determining the competitor concentration at very low levels. By changing the sequence of the competitor DNA, we can readily measure all 3 N single-base mutations of any given consensus sequence of length *N*, typically on a single 96-well plate. A detailed description of the entire experimental and data analysis procedure can be found in the Methods.

### Applying HiP-FA to TFs of the segmentation network

To test our method, we turned to the *Drosophila* segmentation gene network^22,23^, a transcriptional hierarchy that generates the anterior–posterior body plan of the embryo. In total, we tested 21 TFs representing different protein families from this network, plus an additional five TFs from the *Drosophila* ecdysone network. We used the DNA-binding domains (DBD)^5^, and, in one case, full-length TF (Slp1); all proteins were expressed as GST-fusion proteins in *E. coli*. The GST fusion does not alter the results (Supplementary Fig. 3a).

**Fig. 3:**
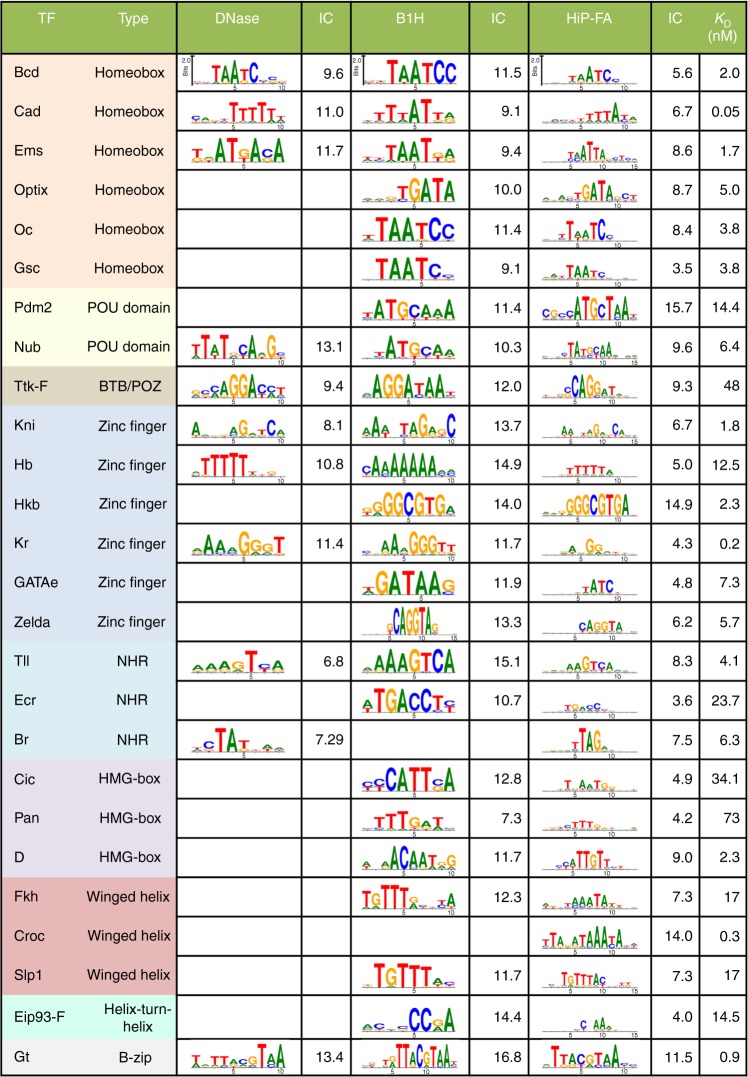
Binding specificities of 26 *Drosophila* transcription factors. The proteins are grouped by type of DNA-binding domains. HIP-FA PWMs are compared with PWMs derived from DNase footprinting^2, 24^ and bacterial one-hybrid (B1H) selection.^8^ Overall, the HIP-FA binding motifs agree with the previous data, but show lower binding specificity combined with high binding affinities. Information content (IC; in bits) and HIP-FA-measured *K*_D_s for the consensus sequences are indicated

As a starting point, we used the homeobox protein Bicoid (Bcd) for a systematic validation of HiP-FA. The Bcd consensus sequence (CGGTAATCCCT) represents the strongest binding sequence, based on previous work^24^, as well as our own HiP-FA (Fig. 1 and Supplementary Data 1). We investigated the influence of all 33 possible point mutations within this 11mer, flanked by additional bases at the 5′ and 3′ end. We measure *K*_D_s ranging from 2.2 nM for the consensus up to 148 nM for sequences with a single-point mutation; we also show near-complete loss of binding for a heavily mutated sequence (Fig. 1f). The binding affinity of Bcd to its consensus is in reasonable agreement with previous measurements (1.14 nM^25^; 3.0 nM^26^), as well as with our own EMSA measurement (*K*_D_ = 1.8 nM, Supplementary Fig. 3b and Supplementary Methods). We also find strong agreement when measuring selected sequences over a range of binding strengths with MST (*r* = 0.96; Fig. 2c and Supplementary Methods; see Discussion). For the entire set of 26 TFs, the *K*_D_s of the consensus sequences range from 50 pM (Cad), 0.9 nM (Gt) to 48 nM (Ttk-F) (Fig. 3), and *K*_D_s for the most detrimental single-point mutation range from 2.2 nM (Cad), 1700 nM (Gt) to ~100 µM (Ttk-F). Thus, single-point mutations can result in a loss in absolute affinity ranging from 44-fold (Cad) to about 2000-fold (Gt and Ttk-F). These data demonstrate that HiP-FA is able to accurately measure binding energies over a very large dynamic range.

We used our data to systematically characterize the binding specificities of the 26 TFs. The DNA binding specificity of a TF is typically represented by a position weight matrix (PWM), which scores the binding strength contribution of every possible nucleotide at every position of the binding site. The standard PWM model has every position contributing independently to the total binding energy and explains the binding preferences of most TFs^27^. Following established procedures^28,29^, we calculated PWMs based on our *K*_D_ measurements; note that as we generate quantitative binding data for each possible point-mutational variant, no motif alignment algorithm is necessary (Methods). We compared them with two types of PWMs, derived from other experimental methods (Fig. 3): one (smaller) set of PWMs is based on aligning all extant binding sites defined by DNA footprinting^2,24^, another set is derived from bacterial one-hybrid (B1H) selection^8^. Overall, the PWMs from the three sets are similar and largely share the same consensus, but the HiP-FA-based PWMs do show significant differences. In the case of Bcd, for example, mutations of T in position 3 of our PWM (Figs. 1g and 3), which is part of the core homeobox binding motif, lead to much stronger binding than expected, given that the previous PWMs show G and A as much less frequently occurring bases. Generally, many of the individual point mutations retain fairly strong binding in our assay; as a result, the HiP-FA-based Bcd PWM is less specific than the previous versions, as is reflected in its lower information content (HiP-FA 5.6 bits<footprinting 9.6 bits<B1H 11.5 bits). This holds true for the larger set of TFs as well: most HiP-FA-derived PWMs are less specific than PWMs derived by other methods, but there are also exceptions, such as Pdm2, Hkb, and Croc, indicating that low specificity is not a generic feature of the assay (Fig. 3). The lower specificity is not unexpected, given that other methods often require stringent washes or selection, while HiP-FA measures equilibrium binding energies over a wide range, allowing it to accurately capture the weaker binding events. Note that we find a weak negative correlation (Pearson coefficient −0.25) between TF affinity (*K*_D_) and specificity, as measured by the information content (IC) of the PWM (Figs. 2d and 3). Thus, factors with more specific binding preferences tend to bind with the DNA slightly more strongly.

### Validation of HiP-FA-derived PWMs

**Fig. 4:**
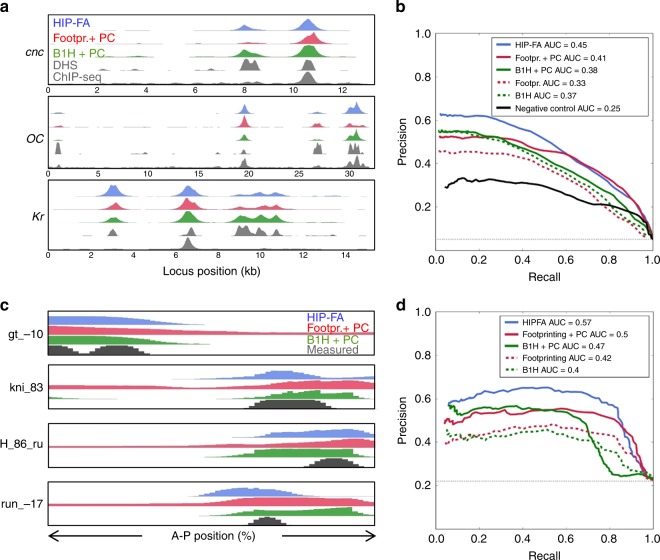
Performance of HIP-FA PWMs (blue), compared to footprinting (red) and B1H (green) PWMs in TF occupancy prediction tasks. The addition of pseudo-counts (PC) is beneficial for footprinting and B1H, but not for HIP-FA PWMs. **a** Prediction of the ChIP-Seq peaks for Bcd in the genomic regions of *cnc*, *oc*, and *Kr*, based on the predicted binding sites and the DNA accessibility (DHS) information, compared to measured ChIP-Seq data. For the prediction, we identified all TF binding sites and their relative strength using PWM and accessibility information, and then applied a gamma function to mimic the fragment length distribution in the ChIP-Seq experiment. **b** Precision–Recall plot for the ChIP-Seq peak predictions over 20 segmentation loci. Each point represents a threshold for the peak prediction; the thin gray line indicates the precision of a random guess. The area under curve (AUC) scores indicate the overall quality of the predictions. **c** Predicted expression patterns based on TF binding site content for four representative segmentation *cis*-regulatory elements, compared with the measured patterns. Shown are relative expression levels from 80% to 20% egg length along the antero-posterior axis of *Drosophila* blastoderm embryos. **d** Precision–Recall plot and AUC scores for the pattern predictions of all 37 *cis*-elements, as in **b**

Given the marked differences between the three PWM sets, we sought to evaluate their performance in explaining the experimental data that measure or reflects in vivo TF occupancy (Fig. 4). We first tested how well the different PWMs can predict experimental ChIP-Seq profiles of five of the TFs where such data are available (Bcd, Cad, Kr, Hb, Gt) in the genomic regions of 21 segmentation genes^20^. Since all PWMs give rise to spurious predictions in regions of closed chromatin, we used DNA accessibility as measured by DNase-Seq as a filter (Fig. 4a and Supplementary Methods)^30^. Chromatin accessibility on its own already shows a moderate correlation with the ChIP-Seq data, but the correlation improves significantly when PWM-based binding site predictions are added. For all five TFs, our HiP-FA-based PWMs score is substantially better in this test than the PWMs derived from B1H or footprinting, showing the highest total correlation (Supplementary Table 2) and best performance in Precision–Recall plots (Fig. 4b). Remarkably, the HiP-FA PWMs remain superior even when pseudo-counts are added to the B1H and the footprinting PWMs, a common practice to globally lower binding specificity. By contrast, the performance of the HiP-FA PWMs does not change significantly with the addition of pseudo-counts, suggesting that their lower specificity is captured accurately. As a second, more complex test, we employed a thermodynamic model^2,31^ that predicts the expression patterns of 37 known enhancers in the segmentation network as a function of the enhancer sequence and of the binding preferences and protein concentrations of the six most important participating TFs (Bcd, Cad, Kr, Hb, Gt, Kni, bold in Fig. 3) (Fig. 4c and Supplementary Methods). We ran the model with the different PWM sets as inputs, while keeping all other inputs constant. Using various metrics, we find that the HiP-FA PWMs consistently outperform the footprinting and B1H PWMs (Fig. 4c, d and Supplementary Table 3), again even when the latter are aided by pseudo-counts. This result is robust against various modifications of the model, such as different objective functions for scoring the agreement between predicted and measured expression patterns, or thresholds for the number of binding site included. An extended model that takes as input 16 of the segmentation TFs similarly shows the HiP-FA PWMs performing substantially better than the B1H PWMs (data not shown).

## Discussion

HiP-FA is a powerful new approach to quantify TF-DNA interactions that overcomes the limitations inherent in most existing methods, and thus represents an important new tool for the quantitative investigation of gene regulatory processes. The method measures equilibrium binding energies directly over a large dynamic range with high accuracy and at large scale. Binding takes place in solution without immobilization of the interacting partners, thus avoiding the limitations of surface-based methods. The controlled delivery system allows the sampling of many different competitor DNA concentrations rapidly within a single well and generating high point-density titration curves, while saving protein and increasing throughput. Throughput is sufficient to measure all single-point mutations of a consensus binding motif within a single 96-well plate and thus derive a PWM that captures the full binding preference landscape of a given TF. A key advantage of this approach is that the strength of the weaker binding events is measured directly—there are no washing or thresholding steps that such binding events have to pass, and there is no need to rely on the assumption that binding strength is reflected in the frequency of nucleotide occurrence in a set of above-threshold binding sites, as is common in most high-throughput methods.

Since it also measures TF-DNA interaction in solution with a highly sensitive readout, MST comes closest to HiP-FA as a method, and we therefore used it to validate our approach. We find excellent correlation between MST and HiP-FA measured *K*_D_s, but observe a noisier MST signal for lower affinities (Fig. 2c). The key drawbacks of MST when measuring TF-DNA interactions are the variable adsorption of the TF protein to the capillary walls, thereby affecting the readout in a non-uniform fashion, and the difficulty in scaling the method to high throughput. Another potentially competitive method is the recently developed SMiLE-seq^14^, a microfluidics-based approach that can identify TF-DNA binding specificities de novo in a semi-high-throughput fashion. SMiLE-seq cannot directly measure the dissociation constants, but relative TF-DNA binding strength is inferred from k-mer enrichments in the library of sequenced bound DNA oligos. We thus compared HiP-FA *K*_D_s with SMiLE-seq k-mer enrichments for the three factors that were measured by both methods (Supplementary Fig. 3c). We found a fair correlation between the two data sets for the strongest binders (*K*_D_ < 20 nM, average Pearson coefficient 0.82), but no correlation for the weaker binders. The weak binders might be partially lost by the washing step applied on the microfluidic device; moreover, the sequencing counts for the weaker binders were typically quite low (<10 counts for 9-mers, data not shown), which means that the Poisson noise is too high to permit accurate quantitation.

The throughput of HiP-FA is not sufficient to permit the de novo discovery of TF binding motifs based on large libraries of random DNA oligos; the method thus requires at least some prior knowledge of a TF’s binding preferences. However, this is not a major limitation, since consensus motifs are known for a large fraction of TFs, and many methods can supply them^5,6,8^. Given a moderate binder, the true maximal binding sequence can be found by simple iteration. To ensure that equilibrium binding is captured, HiP-FA also requires that the TF-DNA binding kinetics be much faster than the diffusion of the competitor DNA through the gel, which occurs within minutes. Similar to other in vitro methods^32^, we find the *K*_OFF_ rates for our TF-DNA interactions to be in the order of seconds and thus sufficiently fast, and in the same range as those measured by other methods^1,27^; the *K*_ON_ rates are much faster (Supplementary Fig. 4). We also checked that the *K*_D_s obtained by HiP-FA are the same as when measured in conventional competitive titration (Fig. 2b and Methods), in which the protein and the DNA are incubated for 1 h to ensure equilibrium (Supplementary Fig. 5). If one seeks to measure interactions with slower binding kinetics, the delivery rate of the competitor in the assay can be retarded by lowering its concentration or reducing the gel pore size. Our data indicate that HiP-FA can measure the dissociation constants reliably over six orders of magnitude, from 10 pM to 10 μM. The lowest *K*_D_ we measured so far was 50 pM (consensus sequence of Cad). The limitation in the high *K*_D_ range only comes from non-specific binding, a known phenomenon for transcription factors; zinc fingers proteins in particular are prone to interact in a non-specific manner with DNA backbones^33,34^.

We have assumed positional independence in constructing our PWMs, limiting the number of sequences that need to be tested. However, we established HiP-FA in 384 as well as 96 multi-well plate format, permitting the measurement of hundreds of DNA sequences in parallel. Therefore, our assay can readily be expanded to measure all possible di-nucleotides or other dependencies within the binding sequence, which may be important for some TFs^5,35,36^, or to measure binding to two neighboring sites. Other DNA features can be assayed as well, such as the role of nucleotides flanking the core binding site or the sequence context more generally. There is no principal limitation regarding the size of the DNA sequences or of the proteins that can be tested. HiP-FA can be implemented by modest adaptation of a standard epifluorescence microscope and can easily be extended to probe other types of molecular interactions, by using the controlled delivery system to generate concentration gradients for other kinds of ligands, such as drugs, peptides, and proteins.

Our approach has generated refined maps for the binding affinity landscapes of 26 TFs, mostly from the *Drosophila* segmentation gene network. We find that their binding specificities cover a wide range, but are in most cases lower than previously measured. Despite their shorter length and lower information content, our PWMs perform significantly better than the PWMs derived from DNA footprinting and B1H data, both in explaining in vivo TF occupancy as measured by ChIP-Seq, and in predicting the spatio-temporal expression of segmentation enhancers. In a computational study, Weirauch et al.^9^ similarly concluded that shorter binding motifs with lower information content improve the prediction of ChIP-Seq data. If individual binding sites carry less information than previously thought, the genome-wide specificity is presumably achieved by local clustering of homotypic or heterotypic sites, i.e., neighboring low-affinity binding sites for the same factor, which also improves robustness against developmental perturbation^37^, or neighboring sequences that allow binding of other TFs and thereby define a combinatorial code^38^. Our findings suggest that this phenomenon is pervasive and a point to the need for a deeper analysis of the genomic context of TF binding sites.

In contrast, the absolute binding affinities of the TFs we measured to their consensus DNA sequence and mutant variants are surprisingly high (Fig. 3). Some of the C2H2 Zn finger proteins show high binding affinity coupled with low specificity suggesting that much of the binding energy derives from interaction with the phosphate backbone of the DNA^33,34^. Most TFs regulate multiple genes in parallel and thus have to bind many different *cis*-element sequences genome-wide; at the same time, many TFs are expressed at relatively low concentrations (100–500 molecules per nucleus^39,40^). Thus, given the relatively low abundance of available TF molecules, high-affinity binding may help ensure that the total occupancy time of TF molecules at their target sites is sufficient to properly effect transcriptional regulation.

## Methods

### Protein expression and purification

For many transcription factors, it is difficult to express full-length proteins at high levels in bacteria or eukaryotic cells^25^. Therefore, we cloned the DNA-binding domains (DBDs) of the TFs (excepted for slp1), flanked by 14 additional amino acids on either side, into the bacterial expression vector, pGEX-6P-1 (GE Healthcare). The polypeptide of interest is fused to an N-terminal glutathione (GST) tag and placed under the control of an IPTG-inducible promoter. The appropriate TF sequences were PCR amplified from either the DGC Gold clone collection (*bcd, cad, kni, hkb, croc, nub, optix, GATAe*; kindly provided by J. Müller), from a TF DNA library (z*elda, slp1, pan, br, ecR, cic;* kindly provided by B. Deplancke) or cDNA prepared from blastoderm embryos (*Kr, hb*, *gt, fkh, ems, gsc, Eip93, oc, pdm2, ttk, tll,* and *D*). The TF-GST fusion constructs were verified by sequencing. The DNA amino-acid sequences of the binding domains can be found in Supplementary Data 1. The fusion constructs were transformed into chemically competent *E. coli* (Top10f, *homemade*), and the protein expression was induced by 1 mM IPTG for 20 h at 18 °C. Incubation at this temperature allows proper protein folding and higher expression levels. The proteins were purified on 5 ml GSTrap columns using an ÄKTA protein purification system (GE Healthcare), following the manufacturer’s protocol. Certain protein preparations contained high levels of bacterial DNA contamination, as judged by the UV spectroscopy (Nanodrop*,*Thermo Scientific), and were therefore subjected to an additional Heparin purification step using 1 ml HiTrapHEP columns. The purity of the proteins was verified by SDS-PAGE. For full-length protein (Slp1), the following modifications were done: the coding sequences were cloned via the gateway method from the entry vectors^41^ into the destination vector that was analogous to the before used pGEX6P1. The plasmids were transformed into *E. coli* BL21-CodonPlus (DE3)-RIPL and expressed via auto-induction^42^ for 4 h at 37 °C and 18 h at 18 °C.

### Controlled delivery assay

The competitive DNA-binding assay is typically performed in 96- or low volume 384-well plate format (175-µm-thick glass bottom; Greiner SensoPlates) (Fig. 1 and Supplementary Fig. 2**)**. Each well contains agarose gel at the bottom and is topped with a competitor DNA, which then diffuses into the agarose gel, thereby forming a concentration gradient whose shape changes over time *c*(*z*,*t*). We checked that the agarose matrix does not lead to any bias in the measurements of the binding energies, e.g., unspecific adsorption to the gel matrix. In the experiment, two different types of wells are used: in the titration or measurement wells, protein and reference DNA labeled with Cy5 are embedded within the agarose gel; Cy5 emits in the far-red range of the visible spectrum and thereby reduces the autofluorescence background. In the calibration wells, only the DNA-intercalating dye Nile Blue is embedded (NB, Sigma). The NB dye binds to the competitor DNA as it diffuses through the gel and thus acts as a concentration sensor. This property is used to determine the concentration of the competitor DNA *c*(*z*,*t*) (Supplementary Fig. 2, for details see below). Typically, three to five calibration wells per plate are enough to determine the DNA concentration accurately, with a CV of <30% between wells.

Once *c*(*z*,*t*) is determined from the calibration wells, the FA in the titration wells can be displayed as a function of concentration (Fig. 1f). These titration curves can then be automatically fitted by our FA software (see below). For competitive assay, the fitting procedure requires the dissociation constant *K*_D1_ for the Cy5-reference DNA-TF interaction, which is obtained separately, by using the same but unlabeled DNA sequence to compete against the labeled DNA in the presence of TF. Different reference DNA sequences will yield the same *K*_D_ values for any given competitor sequence; however, *K*_D_ measurements are most accurate within a range of two orders of magnitude above or below the *K*_D_ of the reference DNA. We therefore typically choose the reference DNA so that its binding affinity falls to the low-to-middle range of the expected *K*_D_s.

### Designing DNA binding sequences and DNA annealing

We seek to measure the *K*_D_ values for all possible single-point mutations of the TF consensus sequence, which is typically 6–10 bp long; flanking bases are added on either side to create DNA oligomers of 16 or 18 bp in length. To establish the consensus, we start from a previously published PWM, e.g., B1H, and test the selected positions with single-point mutations to verify the consensus and to determine the positions that contribute most to the binding energy. In a second round, we then test all 3 N mutations of the true consensus sequence. Note that the secondary binding sites within the DNA oligomer would be recognized during FA measurement and curve fitting, and can be excluded experimentally by finding a non-binding sequence through a more extensive mutation of the consensus. If the initial consensus sequence is incorrect, more mutational iterations may be necessary.

The forward and reverse strands of the DNA oligomers are annealed in water at a concentration of 200 nM for the Cy5-labeled reference DNA and 50 µM for the competitor DNA. The hybridization reactions are performed on a PCR thermal cycler (Eppendorf) by heating up the complementary strands at 70 °C and lowering the temperature to room temperature (RT) (at a ramp of 0.1 K/s).

For the full-length protein (Slp1), interactions of the full-length protein with the fluorescent label of the reference DNA can be a concern depending of the factor. To minimize these interactions, the dye of the labeled reference DNA was changed to BODIPY630 (Eurofins) whose fluorescence proved to be less sensitive to its environment. In addition, the dye was separated from the binding site by a longer spacer of 22 nucleotides. To make oligo synthesis more efficient and economical, a modular system of three oligomers was developed—the sequence containing the binding site was chosen as described above. The spacer sequence with the Bodipy dye covalently attached to its 5′ was chosen in order to avoid any strong binding sites (such as parts of the consensus sequence). A third oligomer complementary to both the reference and the spacer sequence was designed and the DNA was annealed as described above.

### Gel preparation

0.5% w/v low melting temperature agarose (Sigma) is dissolved in the binding buffer (33 mM phosphate buffer pH = 7.0, 90 mM NaCl, and 0.01% Tween 20) at 75 °C. This buffer proved superior to the commonly used Tris binding buffer (see under EMSA), since it increases the stability of the proteins (>10 h at RT) and prevents dimerization of the GST. The agarose gel solution is cooled down to 32 °C. For the titration wells, hybridized Cy5-reference DNA (1 nM), TF protein (*C*_TF_ = 20–60 nM), and DTT (0.1 mM) are added to the 32 °C agarose gel solution, mixed thoroughly, and pipetted into the titration wells of the well plate (200 µl in 96-well plate, 13 µl in 384-well plate). For the calibration wells, NB (5 nM) is added to the 32 °C agarose gel solution, mixed thoroughly, and 200/13 µl are pipetted to the calibration wells of the wellplate.

### Adding the competitor DNA solution

To prevent the formation of concentration gradients of the labeled DNA and the protein within the gel, it is important that the binding buffer, Cy5-reference DNA and TF have the same concentration in the gel and in the competitor DNA solution on top. Therefore, the competitor DNA solutions are diluted 2:1 in a 3× binding buffer containing Cy5-reference DNA (3 nM), TF protein at a concentration 3× *C*_TF_ for the titration wells, and diluted 2:1 in 3× binding buffer with NB (15 nM) for the calibration wells.

A volume of 50 µl (96-well plate) or 7 µl (384-well plate) of the hybridized competitor DNA solutions is added simultaneously with an electronic multichannel pipette (Eppendorf) or with an automated 96-well pipettor on top of the titration and calibration wells. With manual pipetting, the transfer of the gel to the wells should be performed quickly (within less than 2–3 min) to minimize time differences in competitor DNA diffusion between different wells. The plate is then immediately imaged by automated fluorescent microscopy.

### Automation of the gel binding assay

The entire gel binding assay can be prepared manually. However, we transferred the procedure to a Beckmann Coulter Biomek NXp robotic system, which leads to improved accuracy and reproducibility. For example, a critical pipetting step in the assay is the addition of agarose gel solution within the wells of a 96-well plate. The CV for the added volumes improves from 5% with electronic multi-channel pipettes (Eppendorf) to ~1% with the automated system. For the measured dissociation constants, the mean CV improves from <20% with manual pipetting to <15%, and in some cases as low as 5% with automated pipetting in 96-well plate format.

### Experimental setup for microscopy

The FA measurements that are commonly performed on commercial microplate readers, with minimum dye concentration that can be detected with an acceptable signal-to-noise ratio (SNR), is typically >5–10 nM. To accurately measure the high-affinity interactions, such as TF-DNA binding in the nM regime or below, very low-labeled-DNA concentrations have to be used (<1 nM), and determining a single dissociation constant requires a concentration series for the titrating species, using one well per titration point. For all these reasons, it is crucial to have an instrument with improved sensitivity and throughput. Therefore, we built a microscopy setup that can achieve high sensitivity for fluorescence detection as well as fast data acquisition (Supplementary Fig. 1a) and such a setup is capable of measuring FA at different *z*-positions within the agarose gel matrix, permitting to measure an entire titration series within one well.

Our setup is based on an inverted widefield microscope, using a Leica DMI6000 body equipped with a motorized stage, a *z*-piezo stage, and a long distance objective (LEICA HCX PL FLUOAR L 60×/0.60 N.A. Dry). The Cy5-labeled reference DNA molecules are excited at 638 nm with a continuous diode laser (PHOxX 638-40, Omicron, 40 mW) with an intensity of 0.5 kW cm^−2^. The fluorescence is detected on the focal plane of a back-illuminated EM-CCD camera in frame transfer mode (Andor iXon DV897, 512 px × 512 px). Incident laser light is blocked by a dichroic mirror (640 nm cutoff, AHF) and a bandpass filter (ET bandpass 700/75, AHF). For the measurement of FA, a linear polarizer (Thorlabs) is mounted in the excitation path to set the polarization of the excitation light. The fluorescence signal passes behind the emission filter through a polarizing beam splitter (Thorlabs), which splits the emitted light into its perpendicularly and parallel polarized components. These are then focused with an achromatic lens of 200 mm focal length (Thorlabs) on the chip of the camera, and are imaged simultaneously (Supplementary Fig. 1b). The signal-to-noise ratio of FA is typically >10 for concentrations of Cy5-reference DNA solutions as low as 0.1 nM. The detection is highly sensitive due to the use of a high numerical aperture objective, resulting in efficient light collection, and an EM-CCD camera. Thus, small FA changes as low as 10–15 mP can be accurately detected, whereas with a conventional setup, much larger FA changes have to occur in order to be reliably measured. As a result, HiP-FA can monitor binding reactions for which the mass increase is as low as a factor 2. Given that in a typical FA assay the species with the lower molecular weight is labeled (if possible), this implies that HiP-FA is sensitive enough to detect any binding event, whatever the change in mass following the binding reaction.

### Image acquisition

For each well, the time series of *z*-stacks containing 12 fluorescent images of the gel are acquired sequentially at a time resolution of 100–300 ms per frame and with 145 µm step-size, with the lowest *z*-focal plane at a distance of 1400 µm from the coverslip surface. It is important to image so deep into the bulk agarose gel to avoid polarization bias for the fluorescence signal due to the partial back reflection of the emitted light on the coverslip surface. The *z*-stacks are acquired from bottom to top, and generally the measurement of the wellplate is repeated 20–25 time until diffusion of the competitor DNA into the gel is nearly complete. Typically, one cycle is acquired within 5–10 min, depending of the number of wells measured. As the binding kinetics of our TFs are fast (approximately seconds), this measurement time is long enough to ensure the thermodynamic equilibrium. During one cycle, the competitor concentration will differ by less than 30% between the first and the last well, which is sufficiently low not to impede accurate calculation of *K*_D_s. The total measurement time for a wellplate is typically 1.5–2.5 h. Note that, in case of slower binding kinetics or of a larger DNA sequence space, we can slow down the delivery rate of the competitor DNA by either reducing its concentration on top of the gel or by increasing the density of the agarose gel.

### Calculation of the fluorescence anisotropy

Once a wellplate has been imaged, it is necessary to extract the average intensity values for the parallel (*I*^=^) and perpendicularly (*I*^+^) polarized components from the raw fluorescence images of all wells and in an automated fashion. We use a Labview 9.0 (National Instruments) custom-written program (available upon request) that computes the mean pixel intensities from single frames of the two regions of interest (Supplementary Fig. 1b), corresponding to *I*^=^ and *I*^+^. For each well, FA(*z*,*t*) is computed for each *z*-position and time point *t* according to:

1 $${\mathrm {FA}}(z,t) = \frac{{I^ = (z,t) - G \ast I^ + (z,t)}}{{I^ = (z,t) + 2 \ast I^ + (z,t)}}$$

where *G* is the so-called instrument *G*-factor, which corrects for any bias toward the perpendicular channel (for our setup *G* is 1.15).

### Determination of competitor DNA concentrations

We determine the competitor DNA concentration *c*(*z*,*t*) in the gel matrix by using the calibration wells with NB as a sensor for DNA concentration (3–5 calibration wells per wellplate or more depending on SNR). The diffusivity of the competitor DNA in the gel matrix and its affinity to NB depend on the length of the oligomer (different molecular weights), but is independent of its sequence (Supplementary Fig. 2a). Thus, any DNA sequence can be used and *c*(*z*,*t*) corresponds directly to the competitor DNA concentrations in the titration wells, provided that all DNA oligomers have the same number of residues. To determine *c*(*z*,*t*), we first perform a conventional titration series, where NB (5 nM) is embedded in the agarose gel together with different concentrations of the competitor DNA (Supplementary Fig. 2a). The resulting calibration curve is then used to extract *c*(*z*,*t*) from the FA_NB_(*z*,*t*) measurements obtained within the calibration wells on each plate (Supplementary Fig. 2b). However, as the affinity of NB to DNA is relatively weak (*K*_D_=1.7 µM), direct determination of *c*(*z*,*t*) is only possible for *c* > 100 nM. To obtain *c*(*z*,*t*) for *c* < 100 nM, we fitted and extrapolated the concentration profiles *c*(*t*) at a given focal plane *z*, according to Eq. (2) (Supplementary Fig. 2c) This equation is commonly used to calculate one-dimensional sugar concentration gradients, e.g., protein separation.^43,44^ Note that using the fitted function *c*(*z*,*t*) also improves the accuracy for *c*(*z*,*t*) > 100 nM, since it averages out fluctuations in concentration.

2 $$c(z,t) = c_0\left( {1 + {\rm erf}\left( {\frac{{ - z}}{{\sqrt {4D(t + t_0)} }}} \right)} \right)$$

where *c*_0_ is the concentration of the competitor DNA on top of the gel at *t* = 0, erf is the error function, *z* is the position of the focal plane, *D* is the diffusion coefficient of the competitor DNA in the buffer containing agarose gel, and *t*_0_ is the starting time of the measurements. During the fitting procedure, *t*_0_ is kept constant, *c*_0_ and $\frac{z}{{\sqrt D }}$ are used as free parameters.

Eq. (2) assumes free one-dimensional diffusion of the competitor DNA within the gel matrix which, for this purpose can be considered a homogeneous medium, since the gyro-radius of the DNA oligomers (approximately nm) is much smaller than the pore size of the agarose gel (approximately µm). Eq. (2) assumes equal volumes of gel and competitor DNA solution on top; there is no analytical solution when the volumes are different. For our case, Eq. (2) constitutes an approximation of *c*(*z*,*t*) since the volume of the competitor solution is smaller than the volume of the agarose gel in a well (50 µl compared to 200 µl in a 96-well plate; 7 µl compared to 13 µl in a low volume 384-well plate).

### Fitting procedure of the FA titration curves

Our Labview analysis program displays the titration curves obtained by plotting FA(*z*,*t*) as a function of *c*(*z*,*t*) for each individual well and fits them automatically according to the analytical solution, as determined by Roehrl et al.^45^ for competitive fluorescence anisotropy assays:

3 $${\mathrm {FA}} = C \times \frac{{2\sqrt {(d^2 - 3e)} \cos \left( {\frac{\theta }{3}} \right) - d}}{{3K_{{\mathrm {D1}}} + 2\sqrt {(d^2 - 3e)} \cos \left( {\frac{\theta }{3}} \right) - d}} + B$$

with

$$\begin{array}{l}d = K_{\mathrm {D1}} + K_{\mathrm {D2}} + L_{\mathrm {ST}} + L_{\mathrm {T}} - R_{\mathrm {T}}\\ e = \left( {L_{\mathrm {T}} - R_{\mathrm {T}}} \right)K_{{\mathrm {D1}}} + \left( {L_{{\mathrm {ST}}} - R_{\mathrm {T}}} \right)K_{{\mathrm {D2}}} + K_{{\mathrm {D1}}}K_{{\mathrm {D2}}}\\ \quad \quad \quad f = - K_{{\mathrm {D1}}}K_{{\mathrm {D2}}}R_{\mathrm {T}}\\ \quad \quad \quad \theta = {\mathrm {arccos}}\left( {\frac{{ - 2d^3 + 9de - 27f}}{{2\sqrt {(d^2 - 3e)^3} }}} \right)\end{array}$$

*R*_T_ is the total input concentration of the TF, *L*_T_ the unlabeled, and *L*_ST_ the labeled DNA concentration. *K*_D2_ is the dissociation constant of interaction between the two species, *L* and *R*. The dissociation constant *K*_D1_ serves as a reference for the determination of *K*_D2_ and is obtained from the competitive binding of unlabeled DNA oligomer against labeled DNA oligomer of the same sequence in the presence of TF. *C* and *B* are experimental normalization parameters corresponding roughly to the anisotropy hub of the titration curve and to its offset, respectively. Experimentally, *R*_T_ and *L*_ST_ are kept constant, and the relationship between *L*_T_ and FA is analyzed to extract *K*_D2_.

The fitting procedure involves using four free parameters in total: *K*_D2_, *R*_T_, *C*, and *B*. Therefore, as noted before, an important advantage of the competitive assay is the ability to determine *R*_T_, which corresponds to the concentration of active protein, in addition to *K*_D2_. This is of paramount importance when unwanted phenomena, such as protein dimerization or unfolding, might occur that would lead to erroneous estimation of the true (active) TF concentration and thus of *K*_D2_. In fact, for all TFs investigated here, we observe that the concentration of active protein, i.e., TF molecules that actually bind to DNA, is only 40–60% of the total protein concentration, as determined by UV spectrometry. Finally, the software exports, for all titration wells, the parameters obtained during the fitting procedure, in particular the dissociation constant *K*_D2_ and the concentration of active protein *R*_T_.

### Extrapolation of the competitor DNA concentrations

At DNA concentrations lower than 100 nM, we extrapolate *c*(*z*,*t*) from the weakly DNA binding NB dye. To test the validity of this extrapolation, we used DRAQ5 (Biostatus), which has a higher affinity to DNA (*K*_D_~10 nM) as a second dye. We measured the DNA concentrations from 1 to 100 nM and compared them to the extrapolations obtained from NB measurements (Supplementary Fig. 2c). The data points obtained with DRAQ5 align well with the extrapolated *c*(*z*,*t*) curves for *C* < 100 nM, which indicates that NB is sufficient for accurate determination of *c*(*z*,*t*) over a broad range of concentrations. Note that, the DRAQ5 signal is much noisier than the NB signal, since the dye is 10-fold dimmer.

### Competitive titration for very strong DNA binding

In our competitive binding assay, the concentration of the active protein is given by the shift of the FA titration curve along the concentration axis, while *K*_D_ is given by the slope at its inflexion point; steep slopes correspond to low *K*_D_ values. Low *K*_D_ values are thus more difficult to measure, since the point density around the inflexion point is lower and these points are more sensitive to the accuracy with which *c*(*z*,*t*) is determined while diffusing through the agarose gel. In practice, this currently limits the use of agarose gel for delivering competitor DNA to *K*_D_ > 0.5 nM. However, with our HIP-FA setup, we can measure *K*_D_s as low as 50 pM by conducting conventional competitive titrations without agarose gel in a 96-well plate format. By using automation, we serially dilute the unlabeled competitor DNA in a single row of a 96-well plate (0, 1.25, 3.5, 9, 19, 45, 90, 190, 425, 900, 1900, 4000 nM) and added Cy5- or BODIPY630-reference DNA (1 nM) and BCD(-GST) (20-50 nM) at a constant concentration, with a total volume of 200 µl per well in the binding buffer (20 mM Tris-acetate pH 7.0, 50 nM NaCl, 0.01% Tween-20, 0.1 mM DTT). After 40 min, the FA value of each sample is measured and the data points are used to construct equilibrium binding curves that can be fitted with Eq. (3) (Fig. 3b). The K_D_s are determined by conventional competitive titration, and thus the binding specificities are very close to those determined by HIP-FA (Fig. 3a, b). In the current study, the *K*_D_s and the resulting PWMs for the TFs Kr, Cad, and Croc were obtained in this fashion.

### Kinetic measurements of transcription factor-DNA interactions

Dissociation half-times *τ*_off_ were measured using our HiP-FA setup. TF and its corresponding Cy5-reference DNA were mixed at the same concentration as in the HiP-FA assay in 200 µl of binding buffer in a well of a 96-well plate. The laser excitation beam was focused within the medium and a fluorescence time series was measured continuously with a time resolution of 300 ms/frame (FA timetrace in Supplementary Fig. 5a). At *t* = 10 s, the unlabeled competitor DNA solution (strong binder) was quickly added to the medium (*C* = 100 nM) and pipette-mixed thoroughly (green arrow). The abrupt changes in FA observed at *t* > 10 s for all TFs, except for Pan, that was measured using conventional competitive titration (dissociation curves shown for Bcd and Cad in Supplementary Fig. 5a), reveal fast dissociation kinetics within seconds for all studied TFs (Supplementary Fig. 5b).

The dissociation half-times *τ*_off_ were obtained by fitting the FA dissociation curves with Eq. (4) assuming a first-order dissociation kinetic:

4 $${\mathrm {FA}}\,(t) = A + Be^{\frac{{ - (t - t_0)}}{{\tau _{\mathrm {OFF}}}}}$$

where *A*, *B* are constants and *t*_0_ is the time of addition of the competitor DNA.

The dissociation and association rate constants *k*_OFF_ and *k*_ON_ were calculated according to Eqs. 5 and 6, respectively.

5 $$k_{\mathrm {OFF}} = \frac{1}{{\tau _{\mathrm {OFF}}}}$$

6 $$k_{\mathrm {ON}} = \frac{{k_{\mathrm {OFF}}}}{{k_{\mathrm {D}}}}$$

where *k*_D_ is the dissociation constant determined by HiP-FA.

### PWM construction and use of pseudo counts

In addition to determining the absolute affinity of TF to DNA sequence, accurate modeling of its sequence specificity is of central importance, since in vivo the TF has to be able to distinguish the functional sites from the nonfunctional sites within all accessible regions in the genome. Specificity is a measure of how strongly the TF binds to all possible DNA sequences, relative to the consensus. It is typically captured in the form of a PWM^46,47^, which represents the relative preference for a nucleotide at any given position within the binding site. The standard PWM model assumes that each position contributes independently to the total binding strength. The validity of this assumption is the subject of much discussion, but it seems to work reasonably well for most TFs^27^. In practice, PWMs are most often determined by counting the nucleotide frequencies at each position of the aligned binding sites, where what counts as a binding site is determined by methods, such as DNA footprinting or B1H selection, with thresholding being a critical issue. Note that, depending on the length of the experimentally identified binding sequences and the characteristics of the preferred motif, properly aligning the sites can be a difficult task. An alternative approach is to derive a PWM from direct affinity measurements. Under the additivity assumption, one needs to only determine the *K*_D_ of the consensus sequence and of each single base mutation of the consensus. The PWM can then be constructed by,

7 $$p_b = \frac{{\frac{1}{{K_{{\mathrm {D}}b}}}}}{{\mathop {\sum }\nolimits_{b\, \in \left\{ {{{\mathrm {A},\,{\mathrm {C}},\,{\mathrm {G}},\,{\mathrm{T}}}}} \right\}} \frac{1}{{K_{{\mathrm {D}}b}}}}}$$

where *p*_*b*_ is the (inferred) probability of nucleotide *b* at a particular sequence position and *K*_D*b*_ the *K*_D_ measured for the corresponding mutation. The specificity of a PWM can be summarized by its information content, calculated as:

8 $${\mathrm {IC}} = \mathop {\sum}\nolimits_{{\mathrm {pos}}} {\mathop {\sum}\nolimits_{b\, \in \,\left\{ {{{\mathrm {A},\,{\mathrm {C}},\,{\mathrm {G}},\,{\mathrm{T}}}}} \right\}} {{\mathrm {PWM}}_{{\mathrm {pos}}}\left( b \right)\,{\mathrm {log}}_2\frac{{{\mathrm {PWM}}_{{\mathrm {pos}}}\left( b \right)}}{{P_B\left( b \right)}}} }$$

where ${\mathrm {PWM}}_{\mathrm {pos}}\left( b \right)$is the probability of finding nucleotide *b* at position pos in the PWM, and $P_B\left( b \right)$ is the background probability of nucleotide *b*, which we set to 0.25 for all nucleotides.

All HiP-FA derived PWMs are listed in Supplementary Data 1 (average PWMs of 2 or 3 replicates). The B1H PWMs were taken from Noyes et al.^8^. The footprinting PWMs are taken from Schroeder et al.^24^ with the exception of Kni, for which we realigned the known binding sites to better match the consensus established by B1H and HiP-FA; these PWMs are also collected in Supplementary Data 1. The sequence logos for the different PWMs were created using WebLogo 3.0 (http://weblogo.threeplusone.com/create.cgi).

Many methods yield overly specific PWMs, either because they rely on a small number of recorded binding sites, or are produced by a method that is biased toward strong binding events. In this case, weaker potential sites can have a PWM score of zero, which would be highly limiting in binding site prediction tasks. A common remedy is to add pseudo-counts (PC)^48^, which ensure that no binding site has zero probability. In the case of the footprinting and the B1H PWMs, which each typically rely on around 10–30 recorded binding sequences, we add 0.25 to every entry in the PWM, representing one additional unspecific site. When applying PC to the HIP-FA PWMs, we add an uncertainty of 1% in every entry.

### Software availability

The HiP-FA software and test datasets can be downloaded from https://github.com/GeneCenterMunich/HiP-FA

### Data availability

The datasets generated during and/or analyzed during the current study are available from the corresponding author on reasonable request.

## Electronic supplementary material

Supplementary Information

Description of Additional Supplementary Files

Supplementary Data 1
