# Author Correction: True equilibrium measurement of transcription factor-DNA binding affinities using automated polarization microscopy

**DOI:** 10.1038/s41467-019-08556-9

**Published:** 2019-02-05

**Authors:** Christophe Jung, Peter Bandilla, Marc von Reutern, Max Schnepf, Susanne Rieder, Ulrich Unnerstall, Ulrike Gaul

**Affiliations:** Gene Center and Department of Biochemistry, Center for Integrated Protein Science Munich (CIPSM), Ludwig-Maximilians-Universität München, Feodor-Lynen-Strasse 25, 81377 München, Germany

Correction to: *Nature Communications* 10.1038/s41467-018-03977-4; published online 23 April 2018

In the original version of this Article, equation 3 contained a sign error whereby the term RT was added when it should have been subtracted. This has now been corrected in the PDF and HTML versions of the Article.

