# Supplementary material for: True equilibrium measurement of transcription factor-DNA binding affinities using automated polarization microscopy
Source: Nat Commun. 2018 Apr 23;9:1605. doi: 10.1038/s41467-018-03977-4 (PMC5913336; doi:10.1038/s41467-018-03977-4)
Supplement: Supplementary file 1 — Supplementary Information [file 41467_2018_3977_MOESM1_ESM.pdf]

Supporting Information for:

True equilibrium measurement of transcription factor-DNA binding affinities using automated polarization microscopy

Christophe Jung et al.

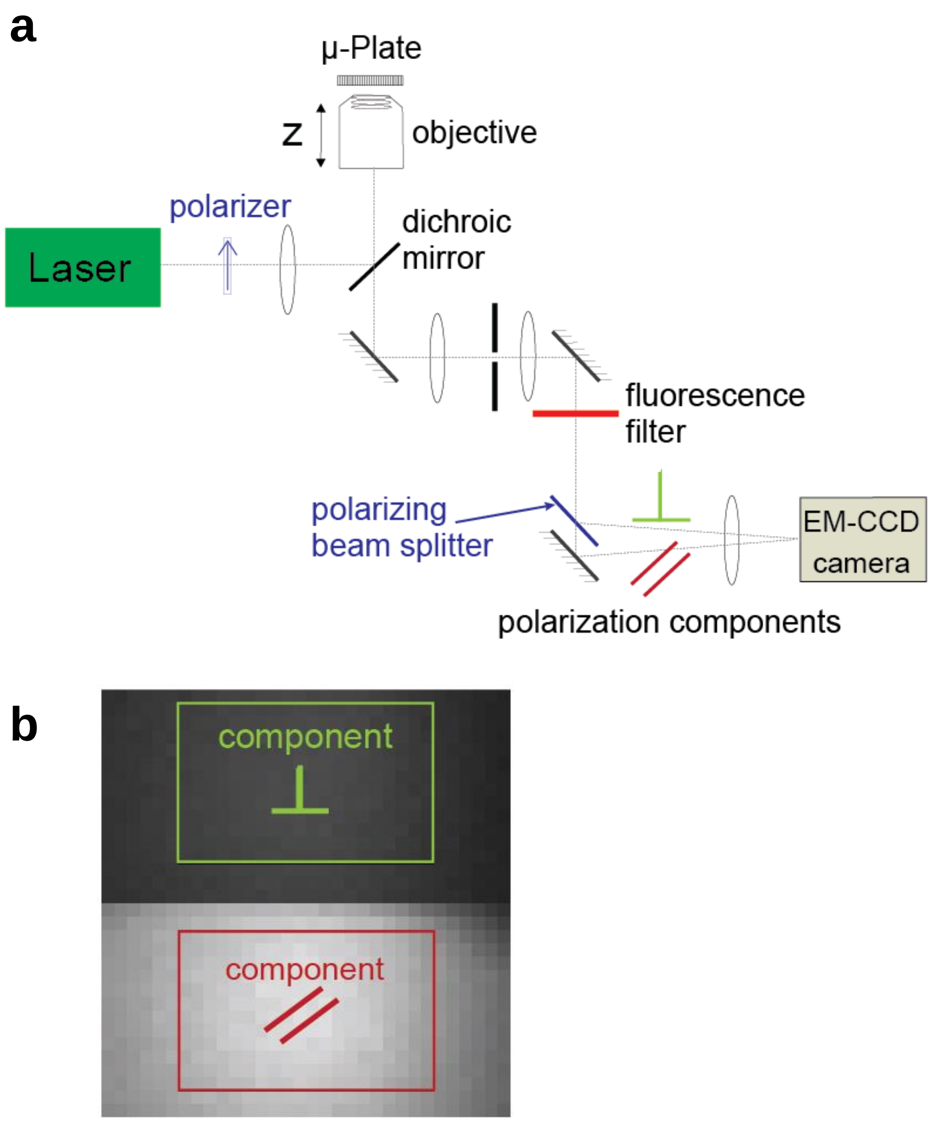

Supplementary Fig. 1. Microscopy setup for the HiP-FA assay. (**a**) Customized automated widefield microscope with polarized fluorescence light detection on an EM-CCD camera. (**b**) Raw fluorescence image exhibiting the parallel (red) and perpendicularly (green) polarized components imaged simultaneously.

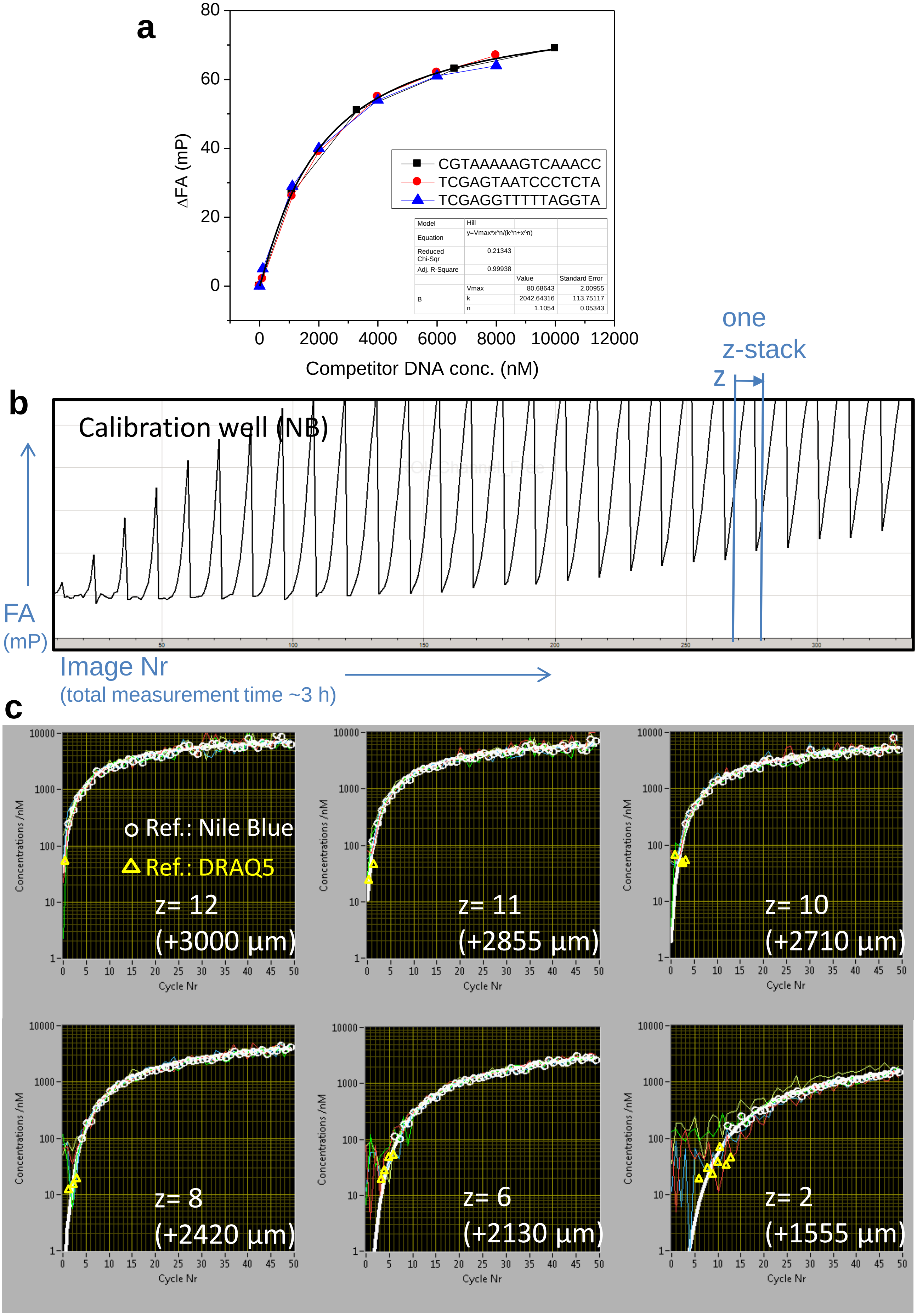

Supplementary Fig. 2

Supplementary Fig. 2. Determining the concentration of the competitor DNA c *(z,t)* using Nile Blue (NB). (**a**) FA-concentration calibration curves for three different 16 bp DNA oligomers. In a conventional titration series, NB (5 nM) is embedded in agarose gel together with different concentrations of competitor DNA. The affinity of NB to DNA is sequence-independent and, therefore, the same calibration curves can be used for determining c*(z,t)* of different DNA sequences of the same length. (**b**) Typical *FA (z,t)* trajectory for a calibration well containing NB (5 nM) in agarose gel and competitor DNA added on top. As competitor DNA diffuses into the gel, it binds to NB, leading to an increase in FA over time. (**c**) Competitor DNA time diffusion profiles for different z heights determined using multiple calibration wells. For each measurement cycle, the average FAs of 4 NB containing wells are displayed as white dots. The curves are fitted using *Equation 2* (white line, **Online Methods**). The average FAs of 4 DRAQ containing wells, measured in separate wells on the same plate, are displayed as yellow triangles. The DRAQ5 data points align with the NB-extrapolated c*(z,t)* curves for $c < 100$ nM, confirming that using the NB dye is sufficient for determination of c*(z,t).*

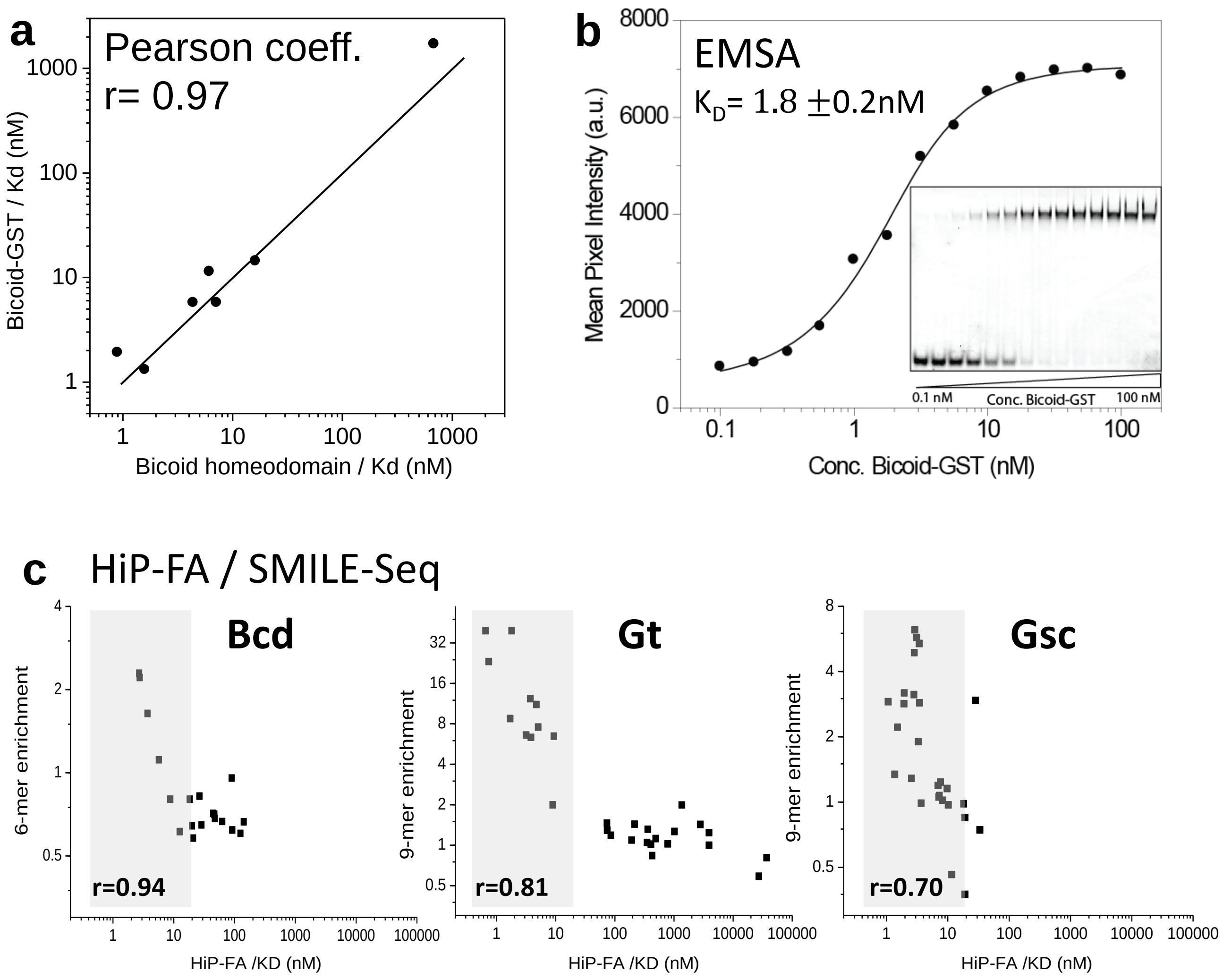

Supplementary Fig. 3. Validation of HIP-FA method. (**a**) Comparison of K_Ds for GST-tagged and untagged Bcd-DBD. Seven different competitor DNAs with single mutations were measured by HIP-FA. (**b**) EMSA with a direct titration of BCD-DBD binding to its consensus. The insert shows the corresponding gel-shift. (c) Correlation between the dissociation constants obtained by HiP-FA and the k-mer SMiLE-seq counts for the three factors that were processed by both methods. The 6-mer enrichment is shown for Bcd, whereas the 9-mer enrichment was used for Gt and Gsc, since these factors have a longer biding site. The r-values denote the Pearson correlation coefficients between the k-mer enrichment and $\log(K_D)$ for strong binders (K_D<20nM, grey box). No correlation is observed for weaker binders, which might be due to the loss of weaker binders during the washing of the microfluidic chip that is required for the SMiLE-seq technique.

a

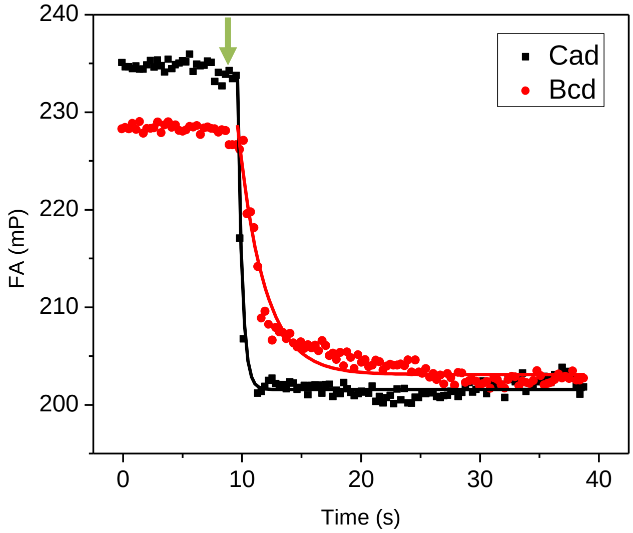

b

TF	T_{off} (s)	k_{off} (s^{-1})	k_{on} ($M^{-1}s^{-1}$)
Bcd	2.5±0.6	0.41	1.9×10^{8}
Cad	0.7±0.5	1.5	3.0×10^{10}
Kr	0.9±0.3	1.2	5.6×10^{9}
Kni	0.7±0.5	1.5	8.5×10^{8}
Hb	1.3±0.5	0.78	6.2×10^{7}
Gt	4.1±0.7	0.24	2.7×10^{8}

Supplementary Fig. 4. Binding kinetics of TF-DNA interactions. (**a**) FA dissociation traces shown with their exponential fits for Bcd and Cad (τ_{off}= 2.5$\pm$0.6s and 0.7$\pm$0.5s). The green arrow indicates the addition of competitor DNA to the solution. (**b**) Kinetic parameters for 6 TFs. The rate constants were calculated as: K_{off} =1 / τ_{off} and K_{on} = K_{off} / K_D. The fast kinetics observed confirm the quick replacement of the Cy5-labeled reference DNA by the competitor DNA (dissociation half-times in the order of seconds), demonstrating that thermodynamic equilibrium is reached in the titration wells during the delivery of the DNA competitor, which takes place over 1.5 - 2.5 hours.

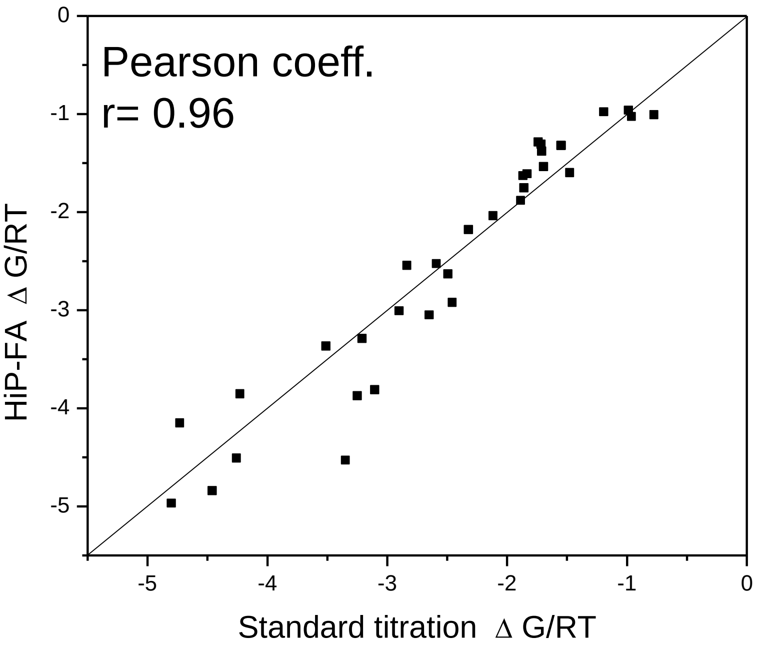

Supplementary Fig. 5. Plot comparing the K_Ds obtained by HiP-FA with the K_Ds obtained by a conventional competitive titration (**Online Methods**). We determined the effects of 33 single point mutations for Bcd both in the agarose gel-based delivery system and by performing a standard titration, via titration series for the competitor DNA solutions. To ensure equilibrium, the solutions were incubated for 1h at RT after mixing TF and DNA. The Pearson correlation coefficient between the K_Ds obtained by the two methods is 0.96. Thus, the two methods result in near identical K_Ds, demonstrating that in the controlled delivery assay, the system is at thermodynamic equilibrium during the delivery of the competitor DNA.

	Acronym	Description	Protein production	[active protein]	Binding kinetics (k_{ON};k_{OFF})	Thermo. equilibrium	K_D range	Sequence space	Labeling	Readout	Quantification of binding preferences	Limitations
**In solution**	**HiP-FA**	Fluorescence anisotropy (FA) measures the rotational diffusion of a fluorescently labeled synthetic DNA oligomer (16-60mers) which decreases upon binding of TF. In HiP-FA, TF and labeled reference DNA are incorporated in a gel matrix, overlaid by a competitive DNA oligomer which diffuses through the gel matrix, forming a spatio-temporal gradient . From the FA(z,t) signal binding affinities are inferred.	Purification from cells	yes	yes	yes	10pM-10µM	10^3	Fluorescently labeled DNA	Fluorescence	Absolute K_D obtained by direct or competitive titration	Medium-throughput, not *de novo*
	**MST**	Measures the motion of molecules along microscopic temperature gradients and detects changes in their hydration shell, charge or size. These parameters change by binding of TF to fluorescently labeled synthetic DNA, (competitive) titration yields binding constants.	Purification from cells	yes	no	yes	pM[1]-mM[2]	10^1	Fluorescently labeled DNA	Fluorescence	Absolute K_D obtained by direct or competitive titration	Low-throughput, protein adsorption on the capillaries, not *de novo*
**Immobiliz-ation of one of the reaction partners**	**MITOMI**	A microfluidic chip is aligned to a glass slide containing micro-arrayed flurorescently labeled synthetic DNA (25 or 70mers) spots. TF is produced by in vitro synthesis directly within the chamber which also contains antibodies to attach the TF to its surface. DNA bound to TF is mechanically trapped and quantified by fluroescence imaging.	*in vitro* synthesis, no purification	no	yes	state close to equilibrium	10nM-10µM[3-5]	10^2 (MITOMI) 10^3 (MITOMI2.0)	Fluorescently and epitope-tagged proteins, fluorescently labeled DNA	Fluorescence	Absolute K_D, direct titration (MITOMI)[3]; or K_D calculated from single-concentration measurement (MITOMI2.0)[4]	Sensitivity, unspecific absorption on the surface, steric hindrance effects, Loss of weak binders due to washing steps, not *de novo*
	**SMiLE-Seq**	A microfluidics-based ligand enrichment technology followed by sequencing. A different TF is mixed with a random dsDNA library in each well of the microfluidic device. Newly formed TF-DNA complexes are trapped similarly to the MITOMI method and unbound molecules are washed away. Bound DNA is recovered, amplified and sequenced.[6]	*in vitro* synthesis, no purification	no	no	state close to equilibrium	1nM-20nM	10^9	Fluorescently and epitope-tagged proteins, fluorescently labeled DNA	Fluorescence followed by sequencing	Specificities	Sensitivity, unspecific absorption on the surface, steric hindrance effects, loss of weak binders due to the washing steps.
	**HiTS-FLIP**	A complex DNA library (25mers) is sequenced in an Illumina sequencing flow cell. Binding of fluorescently tagged TF to the sequenced DNA clusters is visualized using the sequencer optics, affinities can be determined by direct titration.	Purification from cells	no	no	no	10nM-µM[7]	10^9	Fluorescently tagged proteins	Sequencing, Fluorescence	Absolute K_D obtained by direct titration	Sensitivity, unspecific absorption on the surface, steric hindrance effects, based on an obsolete sequencing technology
	**SPR**	Synthetic DNA oligomers are attached to surface, when TF is added and binding occurs, mass accumulates on the sensor surface, leading to an increase in refractive index measured by detecting the light intensity reflected from a prism/metal/water interface from a monochromatic incident beam, until equilibrium is reached. By washing the array, this process is reversed. By repeating this cycle at different concentrations, the k_{ON} and the k_{OFF} rates can be obtained, and affinity can be calculated.	Purification from cells	no	yes	no	nM-µM[8, 9]	10^2	Not required	Reflected light intensity	Absolute K_D obtained by direct titration	Loss of weak binders due to washing steps, unspecific absorption on the surface, steric hindrance effects, binding of low MW protein is difficult to detect, not *de novo*
	**PBM**	TF is added to a DNA microarray consisting of complex library (60mers). After washing, a fluorescent antibody is used to quantify protein binding.	Purification from cells or *in vitro* synthesis	no	no	no	No affinity	10^4-10^5	Epitope-tagged proteins, fluorescently-labeled antibodies	Fluorescence	Relative affinities	Sensitivity, unspecific absorption on the surface, loss of weak binders due to washing steps
	**HT-SELEX**	TF is immobilized on beads and incubated with complex DNA library (40-60mers). After washing, bound DNA is recovered, amplified and used as a new set of ligands in subsequent selection cycles. Bound DNA populations are sequenced after each cycle to infer binding specificities.	Purification from cells	no	no	no	No affinity	10^13	His-tagged proteins	Sequencing	Specificities	Loss of weak binders due to washing steps, sequence amplification bias
	**SELEX-seq**	A pool of synthesized DNA oligonucleotides containing a region of 16 random base pairs is made double stranded and then sequenced using Illumina sequencing, resulting in a first set of reads. EMSAs are performed on the random pool and DNA molecules bound to TFs are isolated and amplified by PCR. This enriched pool is sequenced. The affinity-based selection step is repeated multiple times. Information from earlier and later rounds of selection is combined using LOESS regression to estimate the relative binding affinity.[10]	Purification from cells	no	no	no	Relative affinities	10^13	His-tagged proteins	Sequencing	Relative affinities	Loss of weak binders due to washing steps, sequence amplification bias, relative affinities but with limited dynamical range
***In vivo* method**	**B1H**	A complex DNA library (28mers) is cloned upstream of a weak promoter that drives the expression of a selectable marker. The TF is fused to a subunit of RNA polymerase, such that TF binding recruits RNA polymerase and increases promoter activity. Bacterial survival depends on the strength of expression of selectable marker.	E.coli, no purification	no	no	no	No affinity	10^6-10^8	Proteins expressed as a fusion to a subunit of RNA polymerase	Sequencing	Specificities	Stringency of bacterial selection, subject to position effects and saturation, interference by endogenous TFs.

Supplementary Table 1. Comparison of methods for determining TF-DNA binding specificities

Predicting ChIP-seq data from sequence and DHS

Correlation	Bcd	Cad	gt	hb	Kr	Total
**FA-based**	0.705	0.739	0.604	0.64	0.708	0.6792
**FA-based + PC**	0.705	0.739	0.623	0.639	0.697	0.6806
**B1H**	0.598	0.657	0.442	0.521	0.644	0.5724
**B1H + PC**	0.703	0.707	0.442	0.541	0.706	0.6198
**Footprinting**	0.629	0.614	0.253	0.647	0.643	0.5572
**Footprinting + PC**	0.668	0.664	0.575	0.642	0.728	0.6554
**False PWM**	0.494	0.647	0.534	0.429	0.589	0.5386
**Zero Information PWM**	0.516	0.676	0.536	0.41	0.606	0.5488
**Only DHS data**	0.433	0.564	0.451	0.324	0.51	0.4564

Supplementary Table 2. Correlation between predicted and measured ChIP-Seq signals at 20 segmentation loci. The values are color-coded from red (low correlation) to green (high correlation). Results are calculated separately for five TFs with available ChIP-Seq data, and for different PWM sets, with or without pseudo-counts (PC). The last row shows the correlation between DNA accessibility alone, as measured by DHS, and ChIP-seq data. As negative controls, we predict the ChIP peaks using an incorrect PWM (HIP-FA Kni) or a PWM that contains zero information (every site is a binding site).

Correlation per Enhancer

enhancer	HIP-FA	Footprinting + PC	B1H + PC	HIP-FA – Footpr.	HIP-FA – B1H
btd_head	0.64	0.75	0.92	-0.11	-0.28
cnc_(+5)	0.20	0.20	-0.31	0.00	0.50
D_(+4)	0.77	0.56	-0.66	0.21	1.43
eve_1_ru	0.53	0.50	0.56	0.02	-0.04
eve_37ext_ru	0.83	0.49	0.52	0.33	0.31
eve_stripe2	0.37	0.30	0.18	0.07	0.19
eve_stripe4_6	0.72	0.75	0.66	-0.03	0.06
eve_stripe5	0.87	0.44	0.26	0.43	0.61
ftz_+3	0.32	0.23	0.14	0.08	0.18
gt_(-10)	0.84	0.78	0.84	0.06	0.00
gt_(-1)	0.25	0.72	0.31	-0.47	-0.06
gt_(-3)	0.53	0.82	-0.24	-0.29	0.76
h_15_ru	0.50	0.24	0.29	0.27	0.21
h_6_ru	0.73	0.68	0.57	0.05	0.16
hb_anterior_actv	0.74	0.90	0.90	-0.16	-0.16
hb_centr_&_post	-0.27	-0.50	-0.26	0.23	-0.02
h_stripe34_rev	0.56	0.30	-0.01	0.26	0.56
kni_(+1)	0.67	0.76	0.52	-0.09	0.14
kni_(-5)	0.79	0.70	0.85	0.09	-0.06
kni_83_ru	0.90	0.51	0.75	0.38	0.15
knrl_(+8)	0.64	0.64	0.75	0.01	-0.10
Kr_AD2_ru	-0.38	-0.42	-0.40	0.04	0.02
Kr_CD1_ru	0.88	0.72	0.24	0.16	0.64
Kr_CD2_ru	0.49	0.66	-0.21	-0.17	0.70
nub_(-2)	0.67	0.71	0.83	-0.04	-0.16
oc_(+7)	0.97	0.65	0.92	0.32	0.05
oc_otd_early	0.96	0.61	0.94	0.35	0.02
odd_(-3)	0.82	0.74	0.86	0.08	-0.05
odd_(-5)	0.05	0.38	0.30	-0.34	-0.26
pdm2_(+1)	0.73	0.63	0.92	0.09	-0.20
prd_+4	0.67	0.64	0.71	0.03	-0.04
run_-17	0.61	0.48	0.37	0.13	0.25
run_-9	0.93	0.66	0.83	0.27	0.10
run_stripe1	0.42	0.38	0.39	0.05	0.04
run_stripe3	0.90	0.62	0.87	0.28	0.03
run_stripe5	0.29	0.43	0.48	-0.14	-0.19
slp2_(-3)	0.96	0.78	0.76	0.18	0.19
Average CC	0.60	0.53	0.44	0.07	0.15
**Median CC**	**0.67**	**0.63**	**0.52**	**0.04**	**0.14**

Supplementary Table 3. Correlation scores between measured and predicted expression profiles for every single segmentation enhancer. Columns five and six depict the differences in score. Highlighted in green are the enhancers for which the HIP-FA based prediction scores substantially better than the predictions based on Footprinting and B1H PWMs (threshold: 0.25 score difference). Similarly, red indicates enhancers for which HIP-FA PWMs perform worse than their counterparts.

Supplementary Methods

Electrophoretic mobility shift assay (EMSA)

As a control, we performed EMSA to confirm the previously measured K_D of BCD by a direct titration of the protein (**Supplementary Fig. 3b**). BCD-GST fusion protein was titrated with concentrations ranging from 0.1 nM to 100 nM and incubated with Cy3-labeled (4 nM) 16-mer dsDNA (TCGACCG**TAATCC**CTA, *Eurofins*) for 30 min. Higher BCD-GST concentrations could not be used due to protein aggregation. We note that this is a more general problem, which makes it difficult to use direct titration for measuring TF-DNA interactions with higher K_D values. The samples were loaded in shift buffer (20 mM Tris-acetate pH 7.0, 50 nM NaCl, 0.01% Tween-20, 0.1 mM DTT and 10% glycerol) on a 4.5% polyacrylamide gel and run at 3.5 V/cm for 80 minutes at RT. The gel was read on a Typhoon™ 9000 scanner (*GE Healthcare*) and the intensities of the bands measured using *Image J* and analyzed using *Origin 8.5* (*OriginLab*) software. The dissociation constant was calculated by least squares fit using the Hill Equation

<u>*Equation 9*</u>

$$\phi_B = A\frac{[TF]^n}{[TF]^n + {K_D}^n} + B$$

where ϕ_B is the fraction of bound DNA bound, [*TF*] the TF concentration, K_D the dissociation constant of TF to the DNA sequence, *n* the Hill coefficient and *A* and *B* scaling factors. *n* was set to be *n*=1 (one binding site). The error is given by the fitting procedure (Levenberg-Marquardt algorithm).

Microscale Thermophoresis

To more systematically compare HiP-FA dissociation constants with those obtained with another method, we used Microscale Thermophoresis (MST), a well-established technique for measuring binding energies in solution[11, 12] (**Fig. 2c** and **Supplementary Fig. 3c)**. We sought to compare dissociation constants for the binding of BCD-GST to DNA sequences over two orders of magnitude of K_Ds (2-200 nM) and used a competitive binding assay. This type of assay allows keeping the protein concentration low (which saves protein and avoids formation of protein aggregates) and determining the concentration of active protein. All dsDNA oligomers were first hybridized using a PCR thermal cycler. MST was then performed on a *Monilith NT.45* instrument

(*NanoTemper Technologies*) using hydrophobically coated capillaries (*NanoTemper Technologies*) to avoid protein adsorption to the capillary walls. BCD-GST concentration (50 nM) and Cy3-labelled reference dsDNA concentration (4 nM) were kept constant, while competitor dsDNA was titrated from 1 nM to 4 µM. The data were fitted with *Equation 1* using a custom-written *Labview 9.0 (National Instrument)* script (see Online methods).

Predicting experimental ChIP-seq data

Following Kaplan et al.[13], we developed an algorithm that compares measured ChIP-Seq profiles with simulations based on predicted binding sites. In a first step, the program calculates the binding weight *w* of a TF for every position on both strands of the DNA, based on the PWM for the factor.

Equation 10

$$w = K_{ab} c_{TF} \prod \frac{PWM_i(S_i)}{P_B(S_i)}$$

where S_i is the nucleotide at the ith position of the potential binding site, PWM_i is the ith row of the PWM and P_B is the background probability distribution for nucleotides. We assume a uniform background model with $P_B = 0.25$. K_{ab} and c_{TF} are free scaling parameters that represent the (unknown) absolute binding energy and protein concentration, respectively. For parameter training, they are combined into a single parameter $K = K_{ab}\, c_{TF}$.

In addition to w, we calculate the sum of all weights of overlapping sites w_c, which compete for the binding of TF and cannot be bound simultaneously. The probability that a binding site is occupied is then given by

Equation 11

$$p = \frac{w}{1+w+w_c}$$

In a second step, the algorithm transforms all predicted binding sites into ChIP peaks with relative height p. For the peak shape we use a Gamma distribution as described by Capaldi et al.[14], assuming an average fragment length of 225 bp.[13] Signals from overlapping peaks get summed at every position to yield the predicted ChIP profile.

Most errors in the prediction come from false positive peaks in regions of closed chromatin. Again following Kaplan et al.[13], we therefore used DNase data as a

measure of DNA accessibility[13], and introduce it as a prior to the binding probability. Specifically, we take the average accessibility in the region overlapping the binding site as the probability that the binding site is accessible, p_{acc}; the binding probability then becomes

<u>*Equation 12*</u>

$$p = p_{acc}\frac{w}{1+w+w_c}$$

To perform the test, we used the regulatory regions around 20 *Drosophila* segmentation genes known to be regulated by the TFs, typically 12-30 kb in size, and ChIP-Seq measurements available for five of the TFs (Bcd, Cad, Hb, Kr, Gt)[15]. We train the free scaling parameter K to maximize the correlation between the predictions and the measurement. Tests were performed separately for the five TFs and the three sets of PWMs, with and without PC. Results were evaluated by comparing the correlation coefficients over all segmentation gene loci and using Precision-Recall plots, which contain the same information as Receiver Operating Characteristic graphs, but provide a better visualization in case of highly skewed data with a lot of true negative results.

Predicting expression patterns of *cis*-regulatory elements

As an additional test for the three PWM sets, we scored their performance in predicting the expression of *cis*-regulatory elements that are known to be regulated by the six key TFs under study. These *cis*-elements are typically ~1 kb in length, act largely autonomously, and typically integrate inputs from multiple TFs in a combinatorial fashion; collectively, they generate the characteristic anterior-posterior expression patterns of the segmentation genes. We have previously developed a thermodynamic model that predicts the expression patterns of segmentation *cis*-elements with good accuracy, based on their sequence and the binding preferences (PWMs) and local concentration of participating TFs[16]. The thermodynamic model predicts promoter occupancy, which is assumed to be proportional to the expression level, in a two-step process. In the first step, the model estimates the occupancy of every TF binding site based on its binding preferences, assuming that the system is in equilibrium. In the second step, the model integrates the activatory potential of all binding sites, i.e. the interaction parameters between the TFs and PolII. The final promoter occupancy is

based on a logistic response function to the activatory input of the enhancer. Two parameters determine the role of a TF. The binding affinity is a measure of the binding strength of the TF affecting the site occupancy. Weakly bound sites contribute less to the integrated enhancer response than firmly bound sites. The activatory potential determines the interaction strength between the TFs and PolII and whether a TF is an activator or a repressor. Repressors negate the activatory input of activators. The transition from activators to repressors is continuous and predicting the role of a TF is part of the parameter training.

For the current study, we used the GEMSTAT software package[17]. GEMSTAT calculates the promoter occupancy very efficiently and can take TF binding cooperativity into account. We applied it to 37 segmentation *cis*-elements[16] that are expressed in the trunk region of the embryo (20-80% egg length), where the patterning is best understood. As input we take the anterior-posterior concentration profiles[16] and the different PWM sets for the six TFs (Bcd, Cad, Kr, Hb, Kni, Gt). GEMSTAT has four sets of training parameters: First, the absolute binding affinities, which depend on the unknown absolute concentration of the TFs. Second, the strength of the TF's contribution towards activation or repression. Note that the classification of a TF as an activator or repressor is left to the parameter training. Third, the basal transcription rate, which represents the unknown strength of the interaction between *cis*-elements and core promoter; the parameter acts as a free scaling factor for the expression. And fourth, a cooperativity parameter that captures the synergy between neighboring binding sites of a given TF. These parameters are trained to maximize the Pearson correlation between predicted and measured *cis*-element expression. The model was run separately for the three PWM sets, under a variety of conditions (such as different binding weight threshold, choice of objective function) and parameter constraints, with the HiP-FA PWMs consistently outperforming the other PWMs.

Supplementary References

1. Jerabek-Willemsen, M. et al. MicroScale Thermophoresis: Interaction analysis and beyond. *Journal of Molecular Structure* **1077**, 101-113 (2014).
2. Baaske, P., Wienken, C.J., Reineck, P., Duhr, S. & Braun, D. Optical Thermophoresis for Quantifying the Buffer Dependence of Aptamer Binding. *Angewandte Chemie International Edition* **49**, 2238-2241 (2010).
3. Maerkl, S.J. & Quake, S.R. A systems approach to measuring the binding energy landscapes of transcription factors. *Science* 315, 233-237 (2007).
4. Fordyce, P.M. et al. De novo identification and biophysical characterization of transcription-factor binding sites with microfluidic affinity analysis. *Nature biotechnology* **28**, 970-976 (2010).
5. Rockel, S., Geertz, M., Hens, K., Deplancke, B. & Maerkl, S.J. iSLIM: a comprehensive approach to mapping and characterizing gene regulatory networks. *Nucleic acids research* **41**, e52 (2013).
6. Isakova, A. et al. SMiLE-seq identifies binding motifs of single and dimeric transcription factors. *Nature Methods* **14**, 316-322 (2017).
7. Nutiu, R. et al. Direct measurement of DNA affinity landscapes on a high-throughput sequencing instrument. *Nature biotechnology* 29, 659-U146 (2011).
8. Day, Y.S.N., Baird, C.L., Rich, R.L. & Myszka, D.G. Direct comparison of binding equilibrium, thermodynamic, and rate constants determined by surface- and solution-based biophysical methods. *Protein Science* **11**, 1017-1025 (2002).
9. Castonguay, R. et al. Soluble Endoglin Specifically Binds Bone Morphogenetic Proteins 9 and 10 via Its Orphan Domain, Inhibits Blood Vessel Formation, and Suppresses Tumor Growth. *The Journal of Biological Chemistry* **286**, 30034-30046 (2011).
10. Slattery, M. et al. Cofactor binding evokes latent differences in DNA binding specificity between Hox proteins. *Cell* **147**, 1270-1282 (2011).
11. Duhr, S. & Braun, D. Why molecules move along a temperature gradient. *Proceedings of the National Academy of Sciences of the United States of America* **103**, 19678-19682 (2006).
12. Wienken, C.J., Baaske, P., Rothbauer, U., Braun, D. & Duhr, S. Protein-binding assays in biological liquids using microscale thermophoresis. *Nat. Commun.* **1** (2010).
13. Kaplan, T. et al. Quantitative Models of the Mechanisms That Control Genome-Wide Patterns of Transcription Factor Binding during Early Drosophila Development. *PLoS Genetics* **7**, e1001290 (2011).
14. Capaldi, A.P. et al. Structure and Function of a Transcriptional Network Activated by the MAPK Hog1. *Nature genetics* **40**, 1300-1306 (2008).
15. Bradley, R.K. et al. Binding site turnover produces pervasive quantitative changes in transcription factor binding between closely related Drosophila species. *PLoS biology* **8**, e1000343 (2010).
16. Segal, E., Raveh-Sadka, T., Schroeder, M., Unnerstall, U. & Gaul, U. Predicting expression patterns from regulatory sequence in Drosophila segmentation. *Nature* **451**, 535-540 (2008).
17. He, X., Samee, M.A., Blatti, C. & Sinha, S. Thermodynamics-based models of transcriptional regulation by enhancers: the roles of synergistic activation, cooperative binding and short-range repression. *PLoS Comput Biol* **6** (2010).
