# Supplementary material for: True equilibrium measurement of transcription factor-DNA binding affinities using automated polarization microscopy
Source: Nat Commun. 2018 Apr 23;9:1605. doi: 10.1038/s41467-018-03977-4 (PMC5913336; doi:10.1038/s41467-018-03977-4)
Supplement: Supplementary file 2 — Description of Additional Supplementary Files [file 41467_2018_3977_MOESM2_ESM.pdf]

Description of Additional Supplementary Files

File Name: Supplementary Data 1
Description: Supplementary Data 1 contains the DNA amino acid sequences of the expressed binding domains, all the HiP-FA PWMs, and the fitting parameters for the HiP-FA measurements of two replicates for the Bicoid binding domain.
